# Phase II trial of radiotherapy plus Huachansu in elderly or chemotherapy-ineligible patients with locally advanced esophageal squamous cell carcinoma

**DOI:** 10.1093/oncolo/oyaf325

**Published:** 2025-09-29

**Authors:** Qi Liu, Jingyi Shen, Yun Chen, Jialiang Zhou, Hui Luo, Jiaying Deng, Dashan Ai, Hongcheng Zhu, Shengnan Hao, Kuaile Zhao

**Affiliations:** Department of Radiation Oncology, Fudan University Shanghai Cancer Center, Shanghai 200032, China; Department of Oncology, Shanghai Medical College, Fudan University, Shanghai 200032, China; Shanghai Clinical Research Center for Radiation Oncology, Shanghai Key Laboratory of Radiation Oncology, Shanghai 200032, China; Department of Radiation Oncology, Fudan University Shanghai Cancer Center, Shanghai 200032, China; Department of Oncology, Shanghai Medical College, Fudan University, Shanghai 200032, China; Shanghai Clinical Research Center for Radiation Oncology, Shanghai Key Laboratory of Radiation Oncology, Shanghai 200032, China; Department of Radiation Oncology, Fudan University Shanghai Cancer Center, Shanghai 200032, China; Department of Oncology, Shanghai Medical College, Fudan University, Shanghai 200032, China; Shanghai Clinical Research Center for Radiation Oncology, Shanghai Key Laboratory of Radiation Oncology, Shanghai 200032, China; Department of Radiation Oncology, Affiliated Hospital of Jiangnan University, Wuxi 214000, China; Department of Radiation Oncology, Jiangxi Province Cancer Hospital, Nanchang 330029, China; Department of Radiation Oncology, Fudan University Shanghai Cancer Center, Shanghai 200032, China; Department of Oncology, Shanghai Medical College, Fudan University, Shanghai 200032, China; Shanghai Clinical Research Center for Radiation Oncology, Shanghai Key Laboratory of Radiation Oncology, Shanghai 200032, China; Department of Radiation Oncology, Fudan University Shanghai Cancer Center, Shanghai 200032, China; Department of Oncology, Shanghai Medical College, Fudan University, Shanghai 200032, China; Shanghai Clinical Research Center for Radiation Oncology, Shanghai Key Laboratory of Radiation Oncology, Shanghai 200032, China; Department of Radiation Oncology, Fudan University Shanghai Cancer Center, Shanghai 200032, China; Department of Oncology, Shanghai Medical College, Fudan University, Shanghai 200032, China; Shanghai Clinical Research Center for Radiation Oncology, Shanghai Key Laboratory of Radiation Oncology, Shanghai 200032, China; Department of Radiation Oncology, Fudan University Shanghai Cancer Center, Shanghai 200032, China; Department of Oncology, Shanghai Medical College, Fudan University, Shanghai 200032, China; Shanghai Clinical Research Center for Radiation Oncology, Shanghai Key Laboratory of Radiation Oncology, Shanghai 200032, China; Department of Radiation Oncology, Fudan University Shanghai Cancer Center, Shanghai 200032, China; Department of Oncology, Shanghai Medical College, Fudan University, Shanghai 200032, China; Shanghai Clinical Research Center for Radiation Oncology, Shanghai Key Laboratory of Radiation Oncology, Shanghai 200032, China

**Keywords:** esophageal squamous cell carcinoma, radiation therapy, Huachansu, phase II study

## Abstract

**Background:**

Preclinical studies have demonstrated that Huachansu, a traditional Chinese medicine derived from toad venom, exhibits radiosensitizing properties. This study aimed to explore whether Huachansu plus radiotherapy can improve the tumor control in elderly and chemotherapy-ineligible patients with locally advanced esophageal squamous cell carcinoma (ESCC).

**Methods:**

Eligible patients were randomly assigned at a 1:1 ratio to receive either radiotherapy alone (RT) or combined Huachansu and radiotherapy (Huachansu + RT). The primary endpoint was locoregional control, and the secondary endpoints were overall survival (OS), progression-free survival (PFS), and treatment-related toxicities.

**Results:**

From September 2015 to January 2020, 126 patients who met the eligibility criteria were randomly assigned to the Huachansu + RT group (*n* = 65) or the RT alone group (*n* = 61) from three hospitals. At a median follow-up of 64.8 months (IQR 37.8-78.3), the median locoregional control time was 12.9 months (95% CI 0-27.0) in the Huachansu + RT group and 22.0 months (95% CI 0-52.0) in the RT alone group (HR = 1.35, 95% CI 0.82-2.22; *P* = .235). The median OS time was 15.0 months (95% CI 10.3-19.7) in the Huachansu + RT group and 17.2 months (95% CI 11.3-23.1) in the RT alone group (HR 1.03 95% CI 0.71-1.51 *P* = .868). There was no significant difference between the two groups in the incidence of acute grade 3 or higher adverse events.

**Conclusions:**

Compared with radiotherapy alone, concurrent radiotherapy with Huachansu injection did not improve the locoregional control rate or survival rate in elderly patients or chemotherapy-ineligible patients with locally advanced ESCC. The trial is registered with ClinicalTrials.org, NCT02647125.

Lessons Learned

- Despite preclinical promise, Huachansu, a traditional Chinese medicine, failed to enhance radiosensitivity or survival outcomes in elderly or chemotherapy-ineligible esophageal squamous cell carcinoma (ESCC) patients in this randomized, open-label, phase II trial. Locoregional control and overall survival were comparable between groups, suggesting limited therapeutic synergy under current dosing. Our study did not meet primary endpoint.
- Huachansu combined with radiation therapy (RT) demonstrated acceptable tolerability, with no significant increase in grade ≥ 3 adverse events or treatment-related deaths compared to RT alone. Huachansu plus RT offers a safer alternative for chemotherapy-ineligible populations but requires optimization to balance efficacy.
- The observed discordance between preclinical multitarget activity and clinical efficacy underscores the need for further rigorous clinical evaluation to clarify if and in which specific therapeutic contexts these agents may provide additive value to existing treatment modalities.

## Background

Esophageal cancer predominantly affects elderly populations, with a global median diagnosis age of 67 years; Patients aged 75 years and above account for 19.6% of total esophageal cancer cases, with mortality rates of 28.4% in males and 36.7% in females.^1^ Despite this high incidence, no specific treatment guidelines exist for elderly patients, who often present with comorbidities or decline surgery and chemotherapy. While chemoradiotherapy (CRT) remains the standard nonsurgical treatment for locally advanced esophageal squamous cell carcinoma (ESCC), its dosing regimens are derived from trials excluding patients aged ≥75 years or those with significant comorbidities. Thus, identifying low-toxicity radiosensitizers to enhance radiation therapy (RT) efficacy is critical for this vulnerable population. Preclinical and clinical evidence suggests that certain Chinese herbal medicines exhibit antitumor, radiosensitizing, immunomodulatory, and radioprotective properties.^2^^,^^3^ Huachansu (cinobufotalin), a traditional Chinese medicine derived from *Bufo gargarizans* skin, has been used for millennia. Its active components—indole alkaloids (eg, bufogenin) and steroidal cardiac glycosides (e.g., bufalin, cinobufotalin)—demonstrate antitumor effects by inhibiting proliferation,^4–6^ inducing apoptosis,^7–9^ modulating immunity,^10–12^ and reducing the toxicity of radiotherapy and chemotherapy.^13–16^ Notably, Huachansu is a standard supportive therapy for advanced hepatocellular carcinoma in China.^11^^,^^17^ Studies indicate Huachansu’s potential as a radiosensitizer, mechanistically attributed to its dual capacity to disrupt DNA double-strand break repair pathways and enhance radiation-triggered apoptotic cascades.^18^ Although Huachansu has been explored in esophageal cancer,^19^ its role in radiosensitizing ESCC remains unconfirmed. This randomized, open-label, phase II trial evaluates whether Huachansu combined with RT improves outcomes in locally advanced ESCC compared to RT alone, offering a potential alternative for elderly and chemotherapy-ineligible patients.

## Trial information

**Table:** 

Trial information	
**Disease**	Locally advanced esophageal squamous cell carcinoma
**Stage of Disease/treatment**	Stage II-IVb (stage IV disease only included metastatic lymph nodes in the supraclavicular or celiac trunk area)
**Prior therapy**	No prior regimen
**Type of study**	Phase II
**Primary endpoints**	Locoregional control
**Secondary endpoints**	Overall survival (OS), progression free survival (PFS), Safety profile.

### Additional details of endpoints or study design

This is a randomized, open-label, phase II trial to evaluate the efficacy of combining Huachansu with radical radiotherapy for tumor control in elderly and chemotherapy-ineligible ESCC patients. The study protocol was approved by the Institutional Ethics Committee of Fudan University Shanghai Cancer Center.

### Patients selection and treatment

Pertinent eligibility criteria were as follows: (1) histologically confirmed ESCC with clinical stage II-IVb disease on the basis of the 6th edition of the American Joint Committee on Cancer^20^ (stage IV disease only included metastatic lymph nodes in the supraclavicular or celiac trunk area) and had not received anticancer treatment for ESCC before; (2) ≥75-year old or patients were considered medically intolerant to chemotherapy for internal medicine or other reasons; (3) had an Eastern Cooperative Oncology Group (ECOG) performance status score of 0-2; (4) were expected to tolerate radiotherapy according to the physician’s evaluation; and (5) had a life expectancy >3 months. The exclusion criteria were as follows: (1) previously received systemic therapy or radiotherapy; (2) had another active malignancy in the last 3 years, except cured nonmelanoma skin cancer or cervical dysplasia; and (3) had uncontrolled severe heart disease. Patients were randomly assigned at a 1:1 ratio either to receive radiotherapy with Huachansu injection (Huachansu + RT group) or radiotherapy alone (RT alone group). The total prescribed RT dose was 61.2 Gy to the planning target volume (PTV) in 34 fractions at 1.8 Gy per fraction, 5 days a week for all patients. In the Huachansu + RT group, Huachansu injection (20 mg/m^2^, q.d.) was given 2 h before radiation through intravenous infusion for five days per week during the entire radiation cycle.

### Objectives and assessment

The primary endpoint of the present study was locoregional control, and the secondary endpoints were overall survival (OS), progression-free survival (PFS), and treatment-related toxicities. Locoregional control was measured from the date of treatment initiation to the date of locoregional failure, which was defined as the recurrence or persistence of the primary tumor and regional lymph nodes according to the 6th edition of the American Joint Committee on Cancer. OS was calculated from the initiation of treatment to the date of death or the last follow-up. PFS was measured as the time from the initiation of treatment to the occurrence of progression, the date of death, or the last follow-up. Treatment response was assessed according to the Response Evaluation Criteria in Solid Tumors, version 1.0.^21^ Adverse events (AEs) were assessed using Common Terminology Criteria for Adverse Events‌ (CTCAE) v4.03. Acute toxicities included AEs occurring within 3 months postradiotherapy, while late toxicities were defined as those manifesting >90 days after treatment completion. The safety analysis encompassed all patients receiving at least one treatment.

### Statistical methods

A sample size of 128 (64 patients for each group) was required to accept the hypothesis that an increase in the 1-year locoregional control from 40% in the RT group to 65% in the Huachansu group occurred, with a power of 80% and a standard error of 0.05. An adjustment of 5% for dropouts resulted in a sample size of 134 patients. Survival data were analyzed according to the intention-to-treat population. Survival curves were constructed using the Kaplan‒Meier method and compared with the log-rank test. The Cox proportional hazard model was used to determine hazard ratios (HRs) and confidence intervals (CIs). *Post hoc* subgroup analyses of patients with different general conditions or cancer statuses were performed. Treatment comparisons of categorical variables were made using the χ² test or Fisher’s exact test, as appropriate. All testing was performed at the .05 significance level. The SPSS software package (version 22.0; SPSS Inc., Chicago, IL, USA) was used for all the statistical analyses.

## Drug information

**Table:** 

Drug information	
**Generic/working name**	Huachansu
**Company name**	Anhui Jinchan Pharmaceutical Co., Ltd
**Drug type**	Traditional Chinese medicine
**Drug class**	Traditional Chinese medicine
**Dose**	20 mg/m^2^
**Route**	IV
**Schedule of administration**	
Two hours before radiation for five days per week during the entire radiation cycle for patients in the Huachansu + RT group.	

## Patient characteristics

**Table:** 

Patient characteristics	
**Number of patients, male**	87
**Number of patients, female**	39
**Stage**	II-IVB (AJCC 6th)
**Number of prior systemic therapies: median (range)**	0
**Performance status: ECOG 0 or 1**	ECOG 0: 37 ECOG 1: 81 ECOG 2: 8
**Cancer types or histologic subtypes**	Esophageal squamous cell carcinoma

## Primary assessment method

**Table:** 

Primary assessment method	
**Title**	Locoregional control (the date of treatment initiation to the date of locoregional failure, which was defined as the recurrence or persistence of the primary tumor and regional lymph nodes according to the 6th edition of the American Joint Committee on Cancer)
**Number of patients screened**	130
**Number of patients enrolled**	126, 65 for Huachansu + RT group and 61 for RT alone group
**Number of patients evaluable for toxicity**	126
**Number of patients evaluated for efficacy**	126
**Evaluation method**	RECIST v1.0 criteria on imaging

## General toxicity profile

**Table:** 

	Huachansu + RT group (*N* = 65)				RT alone group (*N* = 61)				
Adverse event	Grade 1-2	Grade 3	Grade 4	Grade 5	Grade 1-2		Grade 3	Grade 4	Grade 5
**Leukocytopenia**	29(44.6)	1(1.5)	0	0	10(16.4)		1(1.6)	0	0
**Neutropenia**	9(13.8)	0	0	0	2(3.3)		0	0	0
**Anaemia**	23(35.4)	0	0	0	14(23.3)		0	0	0
**Thrombocytopenia**	6(9.2)	1(1.5)	0	0	7(11.5)		1(1.6)	0	0
**Hypokalemia**	2(3.1)	0	1(1.5)	0	1(1.6)		0	0	0
**Hyponatremia**	5(7.7)	1(1.5)	0	0	0		0	0	0
**Arrhythmia**	2(3.1)	0	0	0	1(1.6)		0	0	0
**Pneumonitis**	10(15.4)	0	0	2(3.1)	5(8.2)		0	0	1(1.6)
**Esophagitis**	44(67.7)	2(3.1)	2(3.1)	0	39(63.9)		1(1.6)	0	0
**Cough**	38(58.5)	0	0	0	30(49.2)		1(1.6)	0	0
**Fatigue**	32(49.2)	2(3.1)	0	0	25(41.0)		1(1.6)	0	0
**Nausea**	17(26.2)	0	0	0	15(24.6)		0	0	0
**Vomiting**	6(9.2)	0	0	0	7(11.5)		0	0	0
**Anorexia**	17(26.2)	0	0	0	15(24.6)		0	0	0
**Hiccup**	18(27.7)	0	0	0	15(24.6)		0	0	0
**Diarrhea**	3(4.6)	1(1.5)	0	0	2(3.3)		0	0	0
**Dermatitis**	13(20.0)	0	0	0	11(18.0)		0	0	0
**Hemorrhage**	5(7.7)	1(1.5)	0	1(1.5)	0		0	0	1(1.6)
**Hoarseness**	7(10.8)	0	0	0	6(9.8)		0	0	0
**Arthralgia and myalgia**	16(24.6)	0	0	0	10(16.4)		0	0	0
**Weight Loss**	31(47.7)	0	0	0	17(27.9)		0	0	0
**Constipation**	12(18.5)	1(1.5)	0	0	12(19.7)		0	0	0
**Liver dysfunction**	5(7.7)	0	0	0	5(8.2)		0	0	0
**Late pulmonary injury**	19(29.2)	0	0	0	21(34.4)		0	0	0
**Pericardial effusion**	3(4.6)	0	0	0	1(1.6)		0	0	0

Data are presented as No. (%).

## Outcome notes

**Figure 1.**
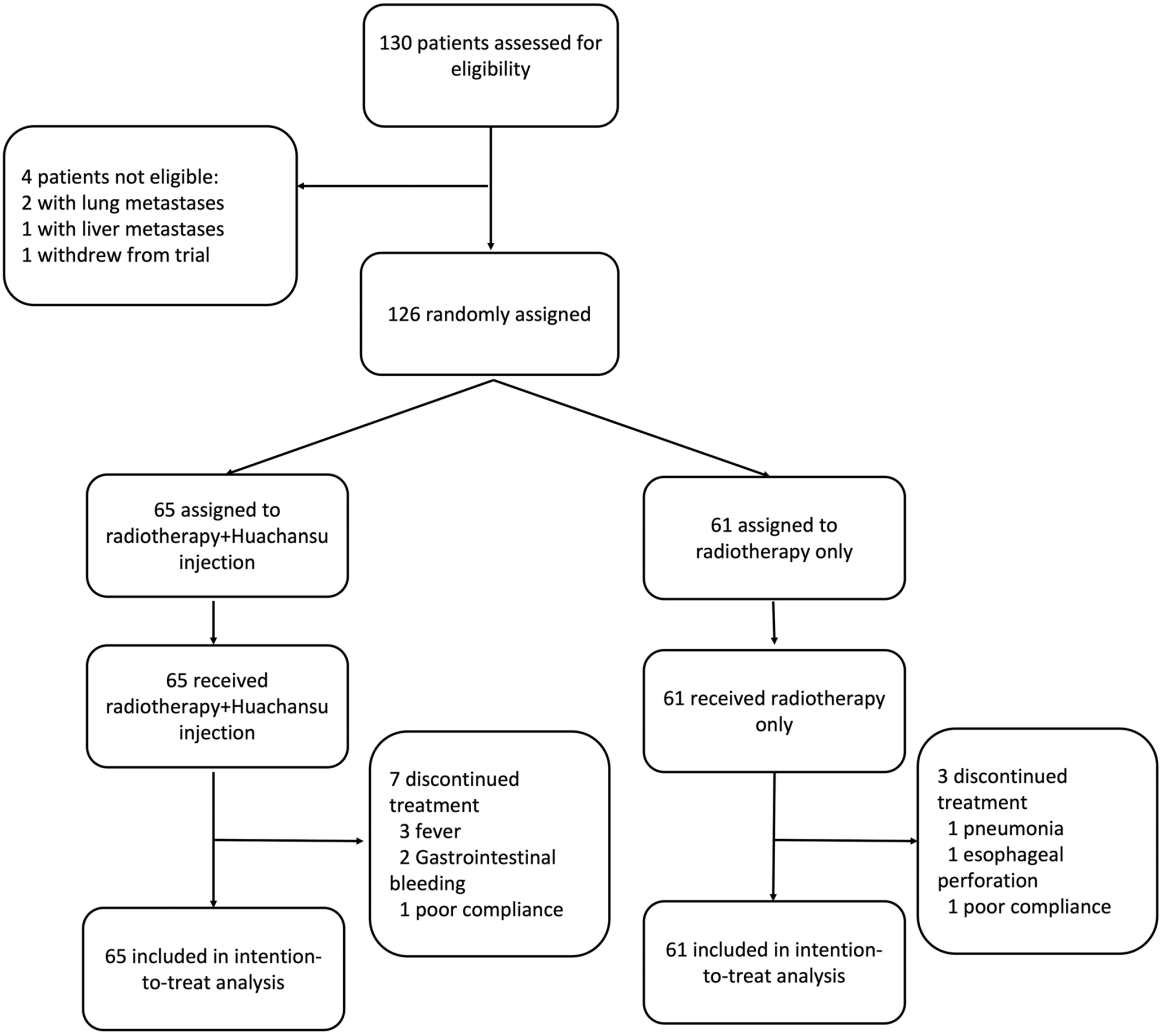
Trial profile.

From September 2015 to January 2020, a total of 130 patients with ESCC were screened from three hospitals in China. Owing to the outbreak of the COVID-19 epidemic since January 2020, screening patients became difficult, so new patient entry stopped at early termination, at which time more than 90% targeted accrual of patients had been achieved (126 of 134 patients). Among them, 65 patients were randomly assigned to the Huachansu + RT group, and 61 patients were randomly assigned to the RT alone group (Figure 1). The baseline characteristics of the two groups were balanced (Table 1).

**Table 1.** Patient characteristics.

Characteristic	Huachansu + RT *N* = 65^a^	RT alone *N* = 61^a^	Total *N* = 126^a^	*P* value
**Age** ^b^	78 (76, 80)	77 (72, 80)	77 (74, 80)	.191
**Sex**				.734
** Male**	44 (67.7%)	43 (70.5%)	87 (69.0%)	
** Female**	21 (32.3%)	18 (29.5%)	39 (31.0%)	
**ECOG score**				.629
** 0**	17(26.2%)	20(32.8%)	37(29.4%)	
** 1**	43(66.2%)	38(62.3%)	81(64.3%)	
** 2**	5(7.7%)	3(4.9%)	8(6.3%)	
**Weight loss**				.305
** 0**	39 (60.0%)	42 (68.9%)	81 (64.3%)	
** >0, <10%**	18 (27.7%)	16 (26.2%)	34 (27.0%)	
** ≥10%**	8 (12.3%)	3 (4.9%)	11 (8.7%)	
**Smoking history**				.938
** No**	36 (58.1%)	35 (57.4%)	71 (57.7%)	
** Yes**	26 (41.9%)	26 (42.6%)	52 (42.3%)	
**Drinking history**				.398
** No**	41 (65.1%)	44 (72.1%)	85 (68.5%)	
** Yes**	22 (34.9%)	17 (27.9%)	39 (31.5%)	
**Dysphagia**				>.999
** No**	12(18.5)	11(18.0)	23(18.3%)	
** Yes**	53(81.5)	50(82.0)	103(81.7%)	
**Site of primary tumor**				.330
** Cervical**	3 (4.8%)	2 (3.3%)	5 (4.0%)	
** Upper and middle thoracic**	40 (63.4%)	45 (73.7%)	85 (68.5%)	
** Lower thoracic**	20 (31.7%)	14 (23.0%)	34 (27.4%)	
** Unknown**	2(3.1%)	0(0)	2(1.6%)	
**Length of primary tumor**				.198
** ≥5 cm**	33 (50.8%)	24 (39.3%)	57 (45.2%)	
** <5 cm**	32 (49.2%)	37 (60.7%)	69 (54.8%)	
**Stage (AJCC 6th)**				.296
** I^c^**	0 (0.0%)	3 (4.9%)	3 (2.4%)	
** IIA**	24 (36.9%)	15 (24.6%)	39 (31.0%)	
** IIB**	7 (10.8%)	9 (14.8%)	16 (12.7%)	
** III**	26 (40.0%)	23 (37.7%)	49 (38.9%)	
** IVA**	3 (4.6%)	6 (9.8%)	9 (7.1%)	
** IVB**	5 (4.6%)	5 (8.2%)	10 (7.9%)	

a *n* (%).

b Median (IQR).

c Three stage I cases were inadvertently enrolled into the RT alone arm due to initial diagnostic misclassification.

### Treatment and safety

All randomly assigned patients received at least one treatment. Among the 65 patients in the Huachansu + RT group, six patients (9.2%) terminated Huachansu early, and five patients (7.7%) experienced Huachansu interruption. Details of Huachansu compliance are shown (Table 2**)**. Sixty-two patients (95.4%) in the Huachansu + RT group and 59 patients (96.7%) in the RT alone group completed at least 50 Gy of irradiation (*P* = .701). The tumor volumes and doses to normal tissues, such as heart and lung, were similar between the two groups (Table 3**)**.

**Table 2.** Details of the Huachansu compliance.

Events	Number	Percent
**Treatment interruption**	5	7.7%
** Gastrointestinal bleeding**	1	1.5%
** Grade 3 thrombocytopenia**	1	1.5%
** Grade 3 fatigue**	1	1.5%
** Treatment for syphilis**	1	1.5%
** Insufficient drug supply**	1	1.5%
**Early termination**	6	9.2%
** Infection**	3	4.6%
** Gastrointestinal bleeding**	2	3.1%
** Intolerance of radiation**	1	1.5%

**Table 3.** Radiotherapy parameters and compliance in randomly assigned patients.

	Huachansu + RT group(*N* = 65)	RT alone group(*N* = 61)
**Radiation parameters**		
**Dose**		
** Completed full dose of planning radiotherapy**	59(90.8)	58(95.1)
** Not completed, but ≥ 50Gy**	3(4.6)	1(1.6)
** Not completed, but <50Gy**	3(4.6)	2(3.3)
**GTV, cm^3^**	35.9 ± 3.8	34.3 ± 4.5
**PTV, cm^3^**	306.6 ± 16.4	294.8 ± 28.9
**Lung V5, %**	55.1 ± 2.0	53.4 ± 1.7
**Lung V20, %**	22.5 ± 2.0	22.7 ± 1.0
**Mean lung dose, Gy**	11.9 ± 0.7	12.2 ± 0.5
**Heart V30, %**	38.4 ± 2.6	32.0 ± 5.5
**Mean heart dose, Gy**	24.7 ± 1.8	21.5 ± 2.9
**Reasons for premature cessation of RT**		
** Treatment-induced toxicities**	3(4.6)	1(1.6)
** Intolerance**	3(4.6)	0(0.0)
** Comorbidity**	0(0.0)	2(3.3)
**Deliver over the planned overall RT time**		
** No delay**	62(95.4)	59(96.7)
** Within 1 week**	3(4.6)	2(3.3)

Data are presented as mean±SD with available data or No. (%). RT: radiotherapy.

There was no significant difference between the two groups in the incidence of acute grade 3 or higher AEs [13 patients (20.0%) in the Huachansu + RT group vs 7 patients (11.5%) in the RT alone group, *P* = .191]. Compared with the RT alone group, the Huachansu + RT group had higher incidences of acute grade 1 or higher leukocytopenia (30 [46.2%] vs 11 [18.0%], *P* < .0001), hyponatremia (6 [9.2%] vs 0 [0%], *P* = .015), and esophageal hemorrhage (7 [10.8%] vs 1 [1.6%], *P* = .036). The incidence rates of late toxicities, such as pulmonary injury (29.2% vs 34.4%, *P* = .531) and pericardial effusion (4.6 vs 1.6%, *P* = .341), were similar between the two groups. Three patients (4.6%) in the Huachansu + RT group and two patients (3.3%) in the RT alone group experienced treatment-related death during or within 3 months after treatment (Table 4).

**Table 4.** Adverse events of patients by treatment group.

	Huachansu + RT group (*N* = 65)				RT alone group (*N* = 61)			
Adverse event	Grade 1-2	Grade 3	Grade 4	Grade 5	Grade 1-2	Grade 3	Grade 4	Grade 5
**Leukocytopenia**	29(44.6)	1(1.5)	0	0	10(16.4)	1(1.6)	0	0
**Neutropenia**	9(13.8)	0	0	0	2(3.3)	0	0	0
**Anemia**	23(35.4)	0	0	0	14(23.3)	0	0	0
**Thrombocytopenia**	6(9.2)	1(1.5)	0	0	7(11.5)	1(1.6)	0	0
**Hypokalemia**	2(3.1)	0	1(1.5)	0	1(1.6)	0	0	0
**Hyponatremia**	5(7.7)	1(1.5)	0	0	0	0	0	0
**Arrhythmia**	2(3.1)	0	0	0	1(1.6)	0	0	0
**Pneumonitis**	10(15.4)	0	0	2(3.1)	5(8.2)	0	0	1(1.6)
**Esophagitis**	44(67.7)	2(3.1)	2(3.1)	0	39(63.9)	1(1.6)	0	0
**Cough**	38(58.5)	0	0	0	30(49.2)	1(1.6)	0	0
**Fatigue**	32(49.2)	2(3.1)	0	0	25(41.0)	1(1.6)	0	0
**Nausea**	17(26.2)	0	0	0	15(24.6)	0	0	0
**Vomiting**	6(9.2)	0	0	0	7(11.5)	0	0	0
**Anorexia**	17(26.2)	0	0	0	15(24.6)	0	0	0
**Hiccup**	18(27.7)	0	0	0	15(24.6)	0	0	0
**Diarrhea**	3(4.6)	1(1.5)	0	0	2(3.3)	0	0	0
**Dermatitis**	13(20.0)	0	0	0	11(18.0)	0	0	0
**Hemorrhage**	5(7.7)	1(1.5)	0	1(1.5)	0	0	0	1(1.6)
**Hoarseness**	7(10.8)	0	0	0	6(9.8)	0	0	0
**Arthralgia And myalgia**	16(24.6)	0	0	0	10(16.4)	0	0	0
**Weight loss**	31(47.7)	0	0	0	17(27.9)	0	0	0
**Constipation**	12(18.5)	1(1.5)	0	0	12(19.7)	0	0	0
**Liver dysfunction**	5(7.7)	0	0	0	5(8.2)	0	0	0
**Late pulmonary injury**	19(29.2)	0	0	0	21(34.4)	0	0	0
**Pericardial effusion**	3(4.6)	0	0	0	1(1.6)	0	0	0

Data are presented as No. (%).

### Efficacy

At the time of analysis on September 19, 2022, median follow-up for surviving patients was 55.0 months (IQR 33.3-76.0) in the Huachansu + RT group vs 64.6 months (IQR 50.3-78.3) in the RT-alone group.

**Figure 2.**
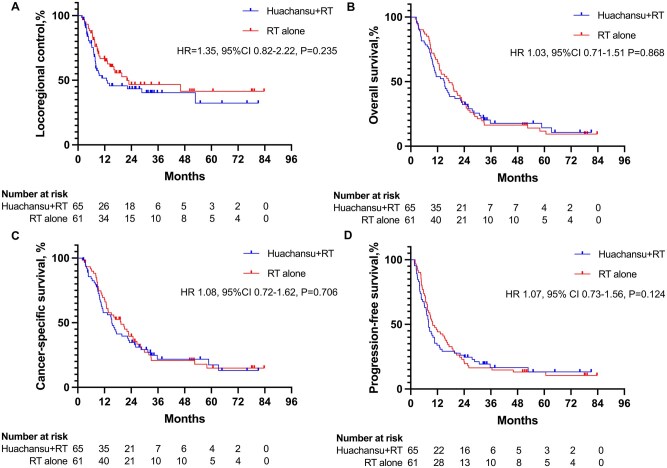
Survival outcomes and cumulative progression incidence in the intention-to-treat population. (A) Locoregional control of the intention-to-treat population. (B) Overall survival of the intention-to-treat population. (C) Esophageal cancer-specific survival of the intention-to-treat population. (D) Progression-free survival of the intention-to-treat population. RT, radiation oncology.

**Figure 3.**
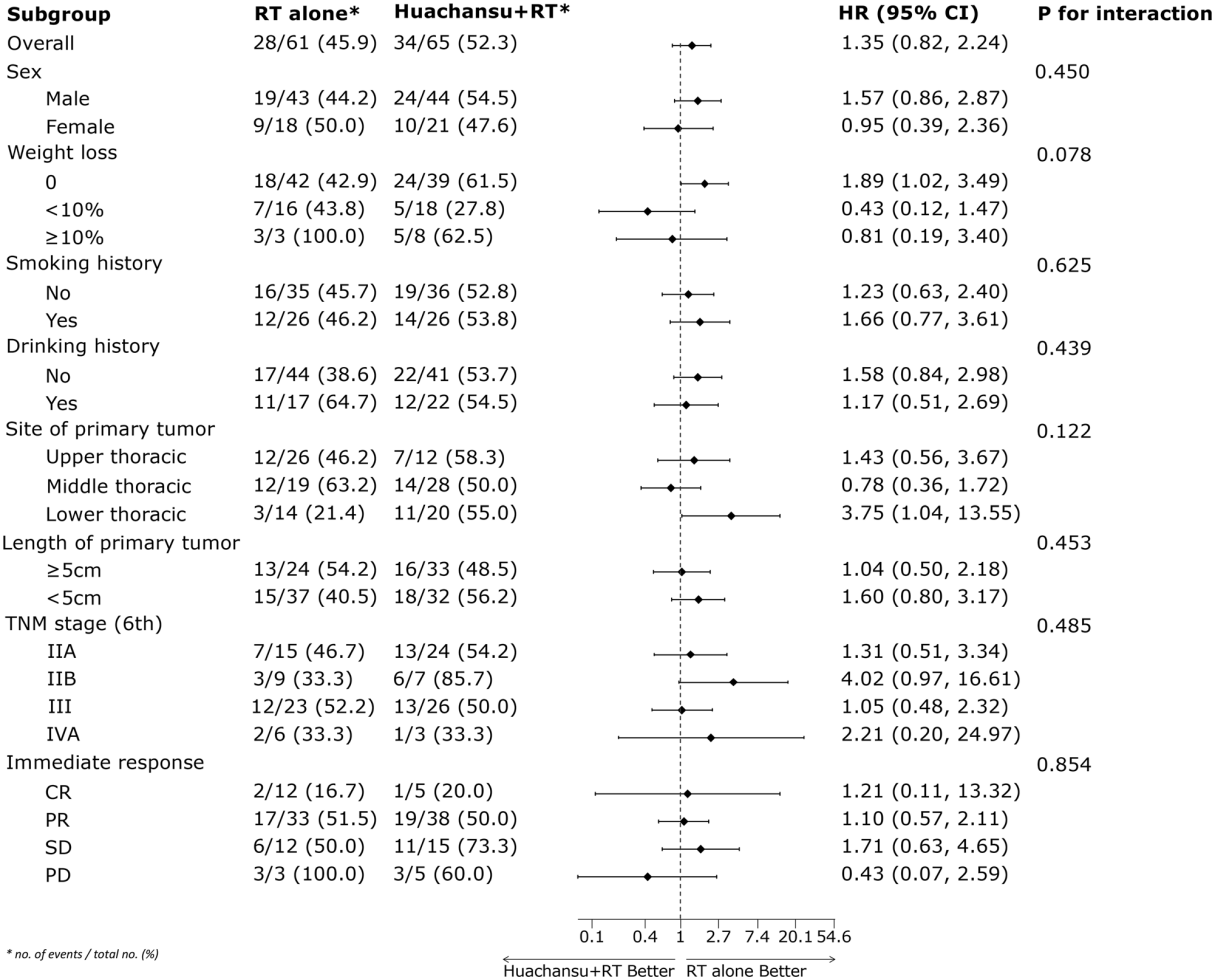
Subgroup analyses of locoregional control in the Huachansu + RT and RT alone groups. RT, radiation oncology. CR, complete response. PR, partial response. SD, stable disease. PD, progressive disease.

The Huachansu + RT group exhibited a median locoregional control time of 12.9 months (95% CI 0-27.0) compared to 22.0 months (95% CI 0-52.0) in the RT-alone group (HR = 1.35, 95% CI 0.82-2.22; *P *= .235; Figure 2A). Cumulative locoregional control rates at 1, 2, 3, and 5 years were 51.7%, 43.4%, 40.3%, and 32.3% for Huachansu + RT vs 64.8%, 46.7%, 46.7%, and 41.5% for RT alone. Locoregional failure occurred in 52.3% (34/65) of Huachansu + RT patients and 45.9% (28/61) of RT-alone patients (Table 5). Overall treatment failure rates were comparable between groups (73.8% vs 75.4%). Subgroup analyses demonstrated no significant intergroup differences in locoregional control across demographic or clinical characteristics (Figure 3). Recognizing the potential impact of the reduced sample size, a *post hoc* power analysis was conducted using PASS 2021 software (v21.0.3). Based on the actual group sample sizes (RT alone: *n* = 61; Huachansu + RT: *n* = 65) and the prespecified effect size of a 25% absolute difference in 1-year locoregional control (40% vs 65%), the achieved post hoc statistical power was 80.969%, which is consistent with the original study design assumption of 80%.

**Table 5.** Patterns of treatment failure.

Type of event in the intention-to-treat population	Huachansu + RT group *N* = 65 (100)	RT alone group *N* = 61 (100)
**Live without treatment failure**	10(15.4)	7(11.5)
**Failure**		
** Locoregional only**	28(43.1)	23(37.7)
** Distant only**	11(16.9)	17(27.9)
** Locoregional and distant**	6(9.2)	5(8.2)
** Second primary tumor**	2(3.1)	1 (1.6)^a^
** Unknown but related with ESCC**	3 (4.6)	2 (3.3)
** Died from other causes^b^**	5(7.7)	8 (13.1)
**Locoregional failure**		
** In-field**	28(43.1)	23 (37.7)
** Out-of-field**	3(4.6)	2(3.3)
** In- and out-of- field**	3(4.6)	3(4.9)

Data are presented as No. (%).

Abbreviation: ESCC, esophageal squamous cell carcinoma.

a One patient occurred both local recurrence in esophagus and second primary esophageal cancer.

b Four patients in Huachansu + RT group and 1 patient in RT alone group died from pneumonia/pneumonitis, one patient in Huachansu + RT group and two patients in RT alone group died as a result of hemorrhage. In RT alone group, two patients died as a result of cerebral infarction, one died as a result of miocardial infarction, one patient died from meningitis, and one patient died as a result of an unknown medical disease.

A total of 108 deaths (85.7%) were recorded, with 83.1% (54/65) in the Huachansu + RT group and 88.5% (54/61) in the RT-alone group. Non-ESCC-related deaths accounted for 7.7% (5/65) and 13.1% (8/61) of cases, respectively. Median OS time was 15.0 months (95% CI 10.3-19.7) vs 17.2 months (95% CI 11.3-23.1; HR = 1.03, 95% CI 0.71-1.51; *P *= .868; Figure 2B). OS rates at 1, 2, 3, and 5 years were 53.8%, 32.3%, 17.8%, and 14.2% for Huachansu + RT vs 65.6%, 34.4%, 16.4%, and 11.7% for RT alone. No significant difference was observed in esophageal cancer-specific survival (median: 15.3 vs 19.4 months; HR = 1.08, 95% CI 0.72-1.62; *P *= .706; Figure 2C). At final analysis, median PFS was 8.0 months (95% CI 6.7-9.3) vs 9.9 months (95% CI 6.4-13.4) (HR = 1.07, 95% CI 0.73-1.56; *P *= .124; Figure 2D). PFS rates at 1, 2, 3, and 5 years were 33.8%, 24.6%, 16.6%, and 13.3% for Huachansu + RT compared to 44.2%, 21.3%, 16.4%, and 13.1% for RT alone.

In the Huachansu + RT group, 36 of 53 patients (67.9%) with baseline dysphagia achieved symptom relief, including 10 (18.9%) with complete resolution. Comparatively, 42/51 patients (82.1%) in the RT alone group showed improvement, with 15 (29.4%) achieving complete symptom remission. Disease progression or stable symptoms were observed in 15 (28.3%) and 6 (11.8%) patients in the Huachansu + RT and RT alone groups, respectively. No statistically significant intergroup difference was observed in dysphagia outcomes (χ^2^=4.793 *P* = .188).

## Discussion

This phase II trial represents the first prospective evaluation of Huachansu combined with radiotherapy in elderly or chemotherapy-ineligible patients with locally advanced ESCC. While the study confirmed the feasibility and acceptable safety profile of Huachansu + RT, it failed to demonstrate therapeutic superiority over RT alone in terms of locoregional control (median: 12.9 vs 22.0 months; HR = 1.35, *P* = .235), survival outcomes (median OS: 15.0 vs 17.2 months; HR = 1.03, *P* = .868), or symptom alleviation. Despite preclinical evidence of Huachansu’s multitarget antitumor activity, clinical translation remains challenging. A prior trial in pancreatic cancer also showed no survival benefit with Huachansu combined with chemotherapy.^22^ These findings warrant critical reflection on the translational challenges of integrating traditional medicine into modern oncology and underscore the unique complexities of managing elderly ESCC populations.

Prior studies have rarely reported locoregional control rates in ESCC.^23–25^ Liu et al.^26^ reported that concurrent CRT achieved a statistically significant reduction in 2-year local recurrence rates (28.1% vs 53.3%; *P* = .018), showing comparable local failure rates observed in our cohort. While Huachansu did not exhibit radiosensitizing or local control benefits, its limited efficacy could indicate either insufficient dosing schedules or a fundamental disconnect between preclinical antitumor activity and clinical applicability—a challenge well-documented in translational research. These findings underscore the necessity of rigorous validation for traditional medicine-derived agents, particularly when preclinical models may not reliably predict clinical outcomes. For elderly or comorbid patients, exploring non-chemotherapeutic alternatives or dose-optimized Huachansu protocols may require further investigation to ­clarify its potential role, if any, in clinical practice.

This study predominantly enrolled elderly patients (80% ≥75 years; median age 77). The RT-alone group demonstrated a median overall survival (OS) of 17.2 months, with 1-/5-year survival rates of 65.6%/8.2%—lower than historical phase III trials using modern radiotherapy (median OS: ∼19-23 months).^23^^,^^24^ This disparity is likely attributable to higher proportions of stage IV (15.0% vs 6.0-12.1%) and IVb (7.9% vs 3.4%-4.8%) disease in our cohort than previous studies, coupled with stricter age inclusion criteria (≥75 vs ≥70 years).^23^^,^^24^ However, several retrospective studies reported 2-year OS rates ranging from 26.0%-35.5% and median OS intervals ranging from 8.6 to 15.2 months in elderly CRT-treated patients,^27–29^ although we cannot directly compare our prospective results with those of retrospective studies. While CRT improves tumor control, survival benefits in elderly populations remain unproven. A 2022 phase II trial reported comparable OS between CRT and RT alone (27.3 vs 19.1 months; *P *= .59),^26^ and Japanese data showed no 3-year OS advantage for CRT in patients >80 years (53.7% vs 59.9%; *P *= .876).^25^ The reason might be that older patients with esophageal cancer generally have an increased risk of toxic effects due to reduced physiologic reserves, as well as a higher prevalence of comorbidities and malnutrition, which lead to an increase in noncancer mortality.^28^^,^^30^

The combination of intravenous chemotherapy and radiotherapy often causes treatment interruption and serious AEs in elderly patients, thereby affecting treatment efficacy.^31^ In contrast, our study demonstrated the safety profile of Huachansu combined with RT for ESCC. The incidence of grade ≥3 acute radiation-related AEs was comparable between groups (13.8% vs 8.2%; *P *= .313). Notably, in the elderly subgroup (≥75 years, constituting 80% of the cohort), Huachansu plus RT did not increase severe AE risks compared to RT alone, demonstrating feasibility without additional safety concerns in this vulnerable population.

This study has several limitations. The premature termination due to COVID-19 reduced the sample size, potentially underpowering subgroup analyses. As this level of post hoc statistical power (80.969%) is consistent with the original study design assumption of 80%, the findings are interpreted as more likely reflecting a truly negative result rather than a consequence of insufficient power. The study lacked comprehensive geriatric, nutritional status and quality-of-life assessments, although we evaluated the patients’ comorbidities and functional status. PET-CT is not a standard examination and is only applicable to patients when available, which may result in inaccurate clinical staging for some patients.

## Data Availability

All authors had access to the data published in this paper. Anonymized dataset may be available from the corresponding author on reasonable request.
